# Current progress achieved in novel materials for supercapacitor electrodes: mini review

**DOI:** 10.1039/c9na00345b

**Published:** 2019-06-27

**Authors:** Sumaiyah Najib, Emre Erdem

**Affiliations:** Sabanci University Nanotechnology Research Centre (SUNUM), Sabanci University TR-34956 Istanbul Turkey emreerdem@sabanciuniv.edu; Faculty of Engineering and Natural Sciences, Sabanci University Tuzla 34956 Istanbul Turkey

## Abstract

Supercapacitors are highly attractive for a large number of emerging mobile devices for addressing energy storage and harvesting issues. This mini review presents a summary of recent developments in supercapacitor research and technology, including all kinds of supercapacitor design techniques using various electrode materials and production methods. It also covers the current progress achieved in novel materials for supercapacitor electrodes. The latest produced EDLC/hybrid/pseudo-supercapacitors have also been described. In particular, metal oxides, specifically ZnO, used as electrode materials are in focus here. Eventually, future developments, prospects, and challenges in supercapacitor research have been elaborated on.

## Abstract 2

This mini-review presents a summary of recent developments in supercapacitor research and technology, including all kinds of supercapacitor design techniques using various electrode materials and production methods for addressing energy storage and harvesting issues.

## Graphical abstract

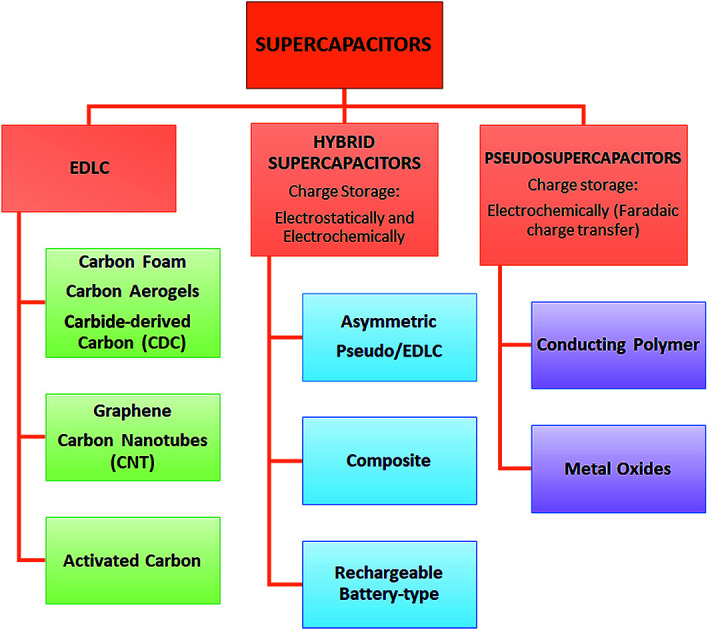

## 1. Introduction

Batteries and capacitors are known as energy storage systems. Batteries are preferred for applications with high energy density but with limited power output requiring long-term use of energy while capacitors are preferred in applications where energy is required to be delivered at high power.

Both batteries and capacitors are insufficient for applications requiring high energy and power density. This leads to an intensive investigation of new types of energy storage systems known as electrochemical capacitors, supercapacitors or ultracapacitors. Supercapacitors are the devices produced to contribute towards finding solutions for the rise in energy demand which is an increasing problem nowadays. The fundamental reason behind such device-development-oriented research is the fact that fossil fuels will soon run out. On the other hand, as important as energy production is, how the energy will be stored, its efficiency and minimizing the environmental concerns are also some of the crucial challenges in this research. Accordingly, supercapacitors have plenty of applications in industrial fields mostly for military purposes and the automotive industry which includes electric vehicles, due to their eco-friendly characteristics, high specific capacitance values, fast charge–discharge peculiarities and promising storage capabilities. Thanks to the tremendous development in supercapacitor technology and research, recently due to their improved energy density values, supercapacitors turned out to be further closer as an alternative than conventional batteries.^1–3^ Indeed, one could easily reach such an ultimate goal where the energy harvesting system can both supply and store high energy and offer high output power by just combining multi-functional advanced materials as electrodes with smarter designs. As mentioned above, it is well known that the energy density of supercapacitors is much lower than that of chemical power sources, but their power density is much higher. The performance of low-cost and eco-friendly energy conversion and storage components which are mostly requirements of electrical energy storage systems, such as batteries and electrochemical capacitors, depends on the physical and chemical properties of electrode materials. It is crucial to create the next generation of energy storage and conversion devices. The pronounced impact of supercapacitors is entirely based on double layers, where the capacitance originates from the pure electrostatic charge accumulated at the electrode/electrolyte interface, and thus the capacitance value strongly depends on the surface area of the electrode materials that is reachable to the electrolyte ions. The capacitance of supercapacitors is defined by electrostatic double layer capacitance or electrochemical pseudocapacitance resulting from reversible reduction and oxidation (redox) reactions or intercalation. A supercapacitor which uses both mechanisms simultaneously is called a hybrid supercapacitor. Additionally, the cycle life of such supercapacitors is extraordinarily long, and they are nearly maintenance-free, have a higher power density, can be charged at high rates, and are much safer than batteries.

The progress of supercapacitor technologies continues mostly towards the development of nanostructured electrode materials. From the morphology point of view, to develop high-performance electrode materials, scientists have designed and integrated one-dimensional (1D) (nanotubes and nanowires), two-dimensional (2D) (nanosheets and nanodiscs), and three-dimensional (3D) nanoarchitectures into electrode materials for the production of supercapacitors and batteries as well. As is seen, both material synthesis and the design of storage and harvesting systems play a vital role in obtaining high-performance from the device. In this mini review we will look at two aspects of supercapacitor research. The first is the material aspect and the second is the design aspect. In the material aspect, we mainly concentrate on the studies which have used ZnO materials as electrode materials. In device application regardless of the electrode materials, we summarize the superior electrochemical performance results of supercapacitor types.

## 2. Electrochemical double layer capacitors (EDLCs)

The working principle of EDLC is based on electrostatically stored charges. The fundamental equation for all capacitors is given as

1 *C* = (*ε*_o_ × *ε*_r_ × *A*)/*d*,

where *A* is the surface area of the electrode; *ε*_o_ is the permittivity of free space; *ε*_r_ is the relative permittivity of the dielectric material; and *d* is the distance between two oppositely biased electrodes.

**Fig. 1:**
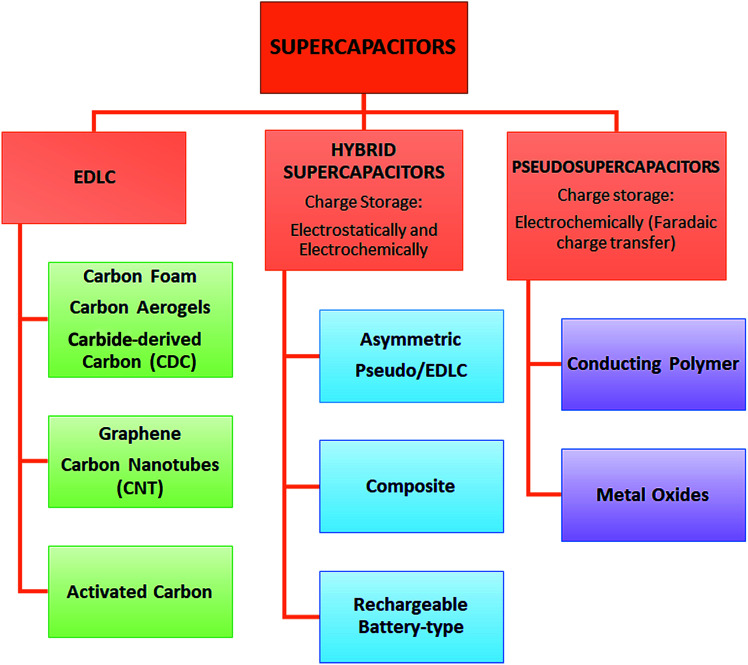
Overview of the types and classification of supercapacitors.

**Fig. 2:**
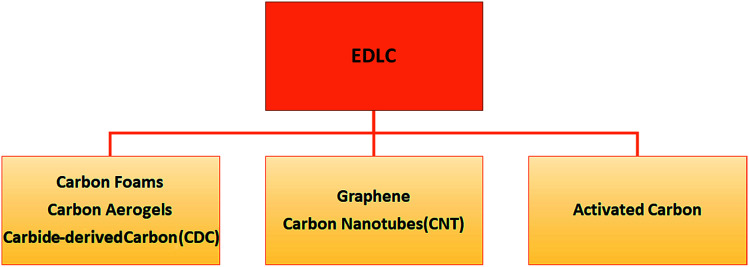
Classification of electrochemical double layer capacitors (EDLCs).

According to the fundamental relationship given in eqn (1), the capacitance of a standard capacitor can be increased by an increase in the dielectric constant of the material and surface area and the decrease of interplanar thickness. However, such an increase can be achieved by further modifying the material system and capacitor design. For instance, one may change the particle size to the sub-nanometer scale where the quantum confinement limits are almost reached. This leads to the material having extraordinary electrochemical performance. Alternatively, metal ion doping *i.e.*, Fe, Mn, Cr, and Co may increase the electrical conductivity of the electrode material which consequently increases the capacitance as well which applies to the design of capacitors. For instance, if a capacitor has symmetric electrodes or its working principle is based on faradaic reactions this supercapacitor can eventually have enhanced electrochemical performance. In short, the faradaic process occurs when Faraday's law is observed, meaning that during this process charge transfer occurs across the electrode–electrolyte interface, whereas in the non-faradaic process, which is addressed after this, Faraday's law is not obeyed *i.e.* charge transfer does not occur, *e.g.* adsorption–desorption at the electrolyte–electrode interface and solvent dipole reorientation. In Fig. 2 the main classification of EDLCs is given that is partly based on Fig. 1.

There are plenty of pros and cons while speaking of EDLC devices that cannot be discussed in detail in a short review article. Therefore, here we concentrate on the most important features. For instance, charging and discharging cycles are highly reversible due to their non-faradaic electrical mechanism. This also gives an extremely stable cycling ability up to 10^6^ cycles or more, a high rate of charging and discharging, and finally little degradation. The main drawback of EDLCs is the limitation in the selection of electrode materials, while in EDLC devices it is necessary to use highly conductive electrodes. Such a shortcoming can be overcome by the recent development of ionic conductive electrolytes. That is why the investigation of ionic electrolytes, in particular solid ones, is a trending hot topic nowadays.^4–6^ There are three main types of EDLCs in terms of the carbon content which leads to different functions or roles in the device. One may distinguish such useful functions by the properties of carbonaceous materials *i.e.* morphology, hybridization and structural defects:

1. Carbon aerogels (nanopores), carbon foams (micropores) and, carbide-derived carbon (CDC) (controllable pore size).

2. Carbon nanotubes (CNTs) and graphene.

3. Activated carbon.

### 2.1 Carbon aerogels, carbon foams and carbide-derived carbon (CDC)

Carbon aerogels are known to be one of the world's lightest materials, with a high surface area and a density of 200 μg cm^−3^.^7^ They display great thermal, electrical and mechanical properties with a high focus on high compressibility and adsorption levels. This has made them a potential successor in the cleaning of any major toxic solution and oil spillage, as they can adsorb liquid oil 900 times their mass.^7^ Although their light-weight structure could be strengthened by a polymer binder, it instead turns into a poor conducting structure. The traditional methods to create amorphous carbon aerogels include sol–gel processes and freeze drying of carbon suspensions. But today, a typical carbon aerogel consists of a fine 3D network of CNTs with an increased Young's modulus *i.e.* super compression and elasticity in addition to its existing mechanical properties.^8^ It has enhanced its mechanical and transport properties, despite its delicate appearance, supported entirely by van der Waals forces between adjacent CNTs. This serves as a prospective lightweight energy storage device, and also, as a potential fine detector of deformation with any slight changes in pressure.^9^ A decrease in its flexibility can result in a carbon aerogel as cracks may emerge during continuous cyclic compression. Different preparation methods allow control of the concentration of the pores independently, making the carbon aerogel a suitable electrode material. However, if the metal precursor is included it results in a change in pH, pyrolysis, activation, *etc.*, making it difficult to manipulate pore consistency.^10^

### 2.2 Graphene and carbon nanotubes (CNTs)

Graphene is a special kind of graphite consisting of a monolayer of carbon atoms of atomic thickness. As it displays outstanding electrical and mechanical properties, it is prevalent and popular through preparations using a variety of techniques. Another reason for its popularity is its ability to include different functional groups into its structure to display a wide array of electrical and mechanical properties. Graphene allows creating flexible structural dimensions whether in 0D, 1D, 2D, and 3D, thus providing a desired finely tuned surface area, enhancing the desired structure. Some different examples include graphene quantum dots, fibre, yarn-like graphene, graphene films, graphene foam and carbon aerogels. Graphene itself is a leading material having high electrical and thermal conductivity, strong mechanical strength and chemical stability. It would be advantageous to embed these properties in constructed supercapacitors along with a high power density and charge/discharge rate and long-life cycle performance.

Graphene can also form numerous nanocomposites with other elements or functional groups to form graphene conductive polymers, graphene with metal oxides, and graphene hydroxide. These nanocomposites allow multiple characteristics to be achieved including a higher specific surface area, higher power density, tractable pore size, electrical conductivity *etc.* However, challenges lie with these composites having their own distinct characteristics, some of which may not contribute to the capacitance or may cause a decrease in overall effective diffusion of ions. Even compromised properties emerge, for instance, a graphene nanocomposite of higher conductivity at the cost of its mechanical strength.^11^ In simple words, a graphene sheet practically rolled up would form a CNT. They are known to be highly conductive. The conduction in CNTs occurs *via* ballistic transport, and this means that charge carriers have a very high mean free path and face no scattering; therefore, no Joule heating takes place. Joule heating or the heating up of conductors while conducting electricity is a major problem in other conductors. But CNTs have a huge advantage in this aspect. CNTs are mainly of two types, namely, single walled nanotubes (SWNTs) and multiwalled nanotubes (MWNTs). SWNTs have a high flexibility and surface area with sizes of around 5 nm. Despite having these highly sought-after properties, they are difficult to handle. This is due to their tendency to get entangled to form bundles. They are cumbersome to isolate and do not share the same properties as an immaculate SWNT. MWNTs on the other hand contain a large number of defects in their structure compared to that of SWNTs. This would result in a change in their structure and ultimately deviating from their intrinsic properties, resulting in higher resistance. But they are longer in size compared to SWNTs, with an average length of 20 nm and have a greater volume than SWNTs and therefore act as an ideal filler material. Although CNTs are claimed to have properties highly sought after, it is their composite forms, that are chemically modified, and reinforced CNT versions that have the finest grade and heightened strengths. Processes utilized in preparing such varied versions of CNTs include solution processing, melt processing, compression moulding, and chemical vapour deposition methods to build modified samples like melt processed fibres, CNT composites, CNT composite fibres *etc.*^12^

### 2.3 Activated carbon (AC)

EDLC devices based on activated carbon electrodes can show superior electrochemical performance not only due to their high surface area but also because of their oxidizing behaviour. Oxidizing carbon materials in general results in the merging or strong overlapping of D and G bands of Raman bands which affects the phonon density of states. This also causes an obvious red shift in Raman lines. Such behaviour can be described by the extensive controlling of pore size, and thus the defect structures such as carbon dangling bonds and C–C bonds. In activated carbon the bonus part is the low cost compared to graphene. Also, activated carbons enable extensive control of pore sizes ranging from below 5 nm to greater than 50 nm. Probably the main drawback of activated carbon electrodes is that the different porous structure does not always contribute to a high capacitance, due to mismatch of electrolyte ion sizes. As a matter of fact, the pore size and ion size of an electrolyte are directly correlated with each other and synergy between these two parameters gives optimum electrochemical performance in a capacitor. Of course, at this point the defect structures of the electrodes play additional crucial roles in the determination of the whole device performance.^13–15^ Thus, to avoid low storage of charge capacity one has to find the best matching of electrolyte ion size and pore size.^16,17^ Recent supercapacitor studies of activated carbon electrode can be found elsewhere.^15,18–20^

## 3. Pseudocapacitors

**Fig. 3:**
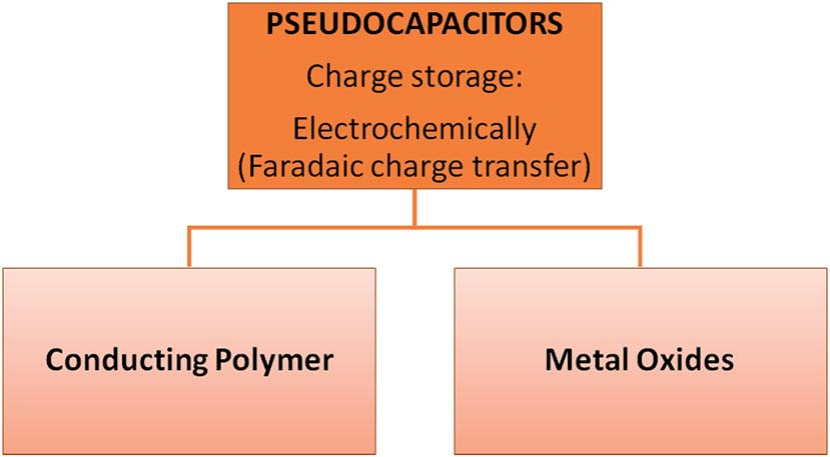
Classification of pseudocapacitors.

These capacitors are faradaic, undergoing redox reactions *i.e.* charge transfer occurs between the electrolyte and electrode. They are prepared using different methods including electrospinning, redox, and intercalation processes. This faradaic process leads to pseudo-capacitors having higher energy densities than EDLCs. Most electrode materials of this type of capacitor include metal oxides, metal-doped carbon and conductive polymers (Fig. 3).^21^ However, pseudocapacitors also have a shorter life cycle and power density. These results occur due to the redox reactions in the capacitors.^22^

### 3.1 Conducting polymer pseudocapacitors

These types of supercapacitors display high capacitance, high conductivity *i.e.* low ESR and low cost compared to carbon based EDLCs. They are said to have great potential densities and some example materials include polypyrrole, polyaniline, and polythiophene.^16^ Attributable to their flexibility and conductivity they are commonly used to improve capacitance as a nanofiller with reportedly higher areal capacitance than EDLCs.^23^ Polymer based electrodes normally have lower cycle stability than carbon based electrodes. When doped, polymers can boost conductivity on one hand though they also display a change of volume, causing swelling and an increase in the thickness of the electrode, which is a hazard for any device.^23^

### 3.2 Metal oxide pseudocapacitors

These materials provide very high conductivity. One of the most researched metal oxides is RuO_2_. It also has low ESR and very high specific capacitance. But its high cost compared to other transition metal oxides has caused researches to diversify into alternate possibilities. However, metal oxides are yet to achieve their potential capacitance values. Some of the processes included in their fabrication process include insertion, intercalation, sol–gel, anodic deposition, spray deposition, hydrothermal synthesis, oxidative synthesis *etc.*^24^ Metal oxides themselves are claimed to provide high capacitance with high energy at a low current density. However, metal oxides are said to cause cracking of electrodes leading to short term stability, as their pores cannot be designed or altered in any form.^25^ In general, they are combined with carbon to form composites to counterbalance these traits. In addition, carbon-based materials are mostly used as the electrode materials for designing pseudocapacitor electrodes and their combination with nanosized transition metal oxide materials was shown to have potential to achieve ultrahigh values of specific capacitance such as Co_3_O_4_, MnO_2_, Fe_3_O_4_ and ZnO. In this review, the synergetic effects of ZnO nanocrystals and the counter electrode will be summarized in detail. More about it can be read in the section about composite hybrid supercapacitors. And yet, there are claims that metal oxides have higher capacitance than carbon-based and conducting polymers.^16^

## 4. Hybrid supercapacitors

**Fig. 4:**
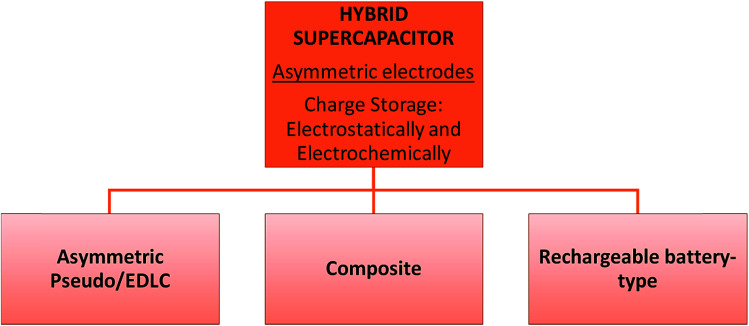
Classification of hybrid supercapacitors into three types according to their design or working mechanism.

This type of supercapacitor consists of polarizable electrodes (carbon) and non-polarizable electrodes (metal or conducting polymer) to store charges. It uses both faradaic and non-faradaic processes^16^*i.e.* making use of these properties to obtain high energy storage through both the battery type and the capacitor type electrode^24^ resulting in better cycling stability and lower costs than EDLCs. It has three main categories: asymmetric, composite, and battery-type as illustrated in Fig. 4.

### 4.1 Asymmetric hybrid supercapacitors

Amongst other supercapacitors, this type is distinctive because of its two dissimilar electrodes. They are designed to work simultaneously to address the power and energy density requirement, as one works as a capacitive electrode and the other as a faradaic electrode. Mostly carbon derived materials serve as the negative electrode while a metal or metal oxide electrode serves as the anode. Metal electrodes are said to have high intrinsic volumetric capacity leading to an increase in the energy densities.^26^ These types of capacitors have the potential to display higher energy density and cycling stability than symmetric supercapacitors. This is the case seen for carbon and MnO_2_ on a nickel foam based electrode.^27^ Self-discharge of a capacitor is a major issue in all capacitors. One way to tackle it is to incorporate in an asymmetric capacitor the simple rocking-chair mechanism. Here is where the maximum potential is ensured at zero current.^26^ It is observed overtime with nearly all electrolytes a depletion of ions and electrodes causes a decrease in the conductivity along with an increase in the internal resistance, and the newly designed electrolytes could feasibly evade this pressing concern. Another challenge is to increase the working voltage of supercapacitors. If the negative electrode is activated carbon or a carbon derived sample effectively p-doped, it has the potential to improve the voltage range along with the rocking-chair mechanism. The carbon microporous layout is also a critical feature to be perfected. Once amended it would allow convenient ion transport to increase the capacitance of the device.^26^

### 4.2 Composite hybrid supercapacitors (CHSs)

The purpose of composite hybrid supercapacitors is to have synergistic outcomes of specific capacitance, cycling stability, and high conductivity. As seen in EDLCs above, carbon based supercapacitors have a great surface area, no Joule heating, low resistance, and great mechanical strength. However, carbon itself shows a poor energy density compared to commercially used lead acid batteries and lithium ion batteries, whereas, metal oxides, which are being investigated, have poor conductivity, with the exception of RuO_2_, and experience Joule heating, with a poor surface area and poor structural stability when under strain. But they are competent in storing charge, and thus energy. Composite hybrid supercpacitors combines the properties of both carbon and metal oxides, merging to provide the synergistic characteristics that are sought after, which include specific capacitance, cycling stability, and high conductivity. Together carbon will provide a channel for charge transport and the metal oxide will store charge *via* redox reactions contributing to high specific capacitance and high energy density. While the conductivity of the composite is highly tunable, it depends on the structure of the carbon whether microporous, or, mesoporous and or, macroporous,^28^ which means that the pore diameter is an essential factor to consider as it determines whether the ions will or will not be adsorbed to the surface of the electrode, defining its EDLC characteristic for charging/discharging. If not, it will show low conductivity. There are limitations of the composites as well for example when vanadium oxide was layered on carbon nanofibers, the efficiency started to decrease when the layer became thicker (18 nm).^29^ This has arisen due to an imbalance between the redox site and conductivity of the composite itself. Also, another challenge arises when the successive ion diffusion declines, due to protruding nanowhiskers that grow on the carbon nanofibres, even though the surface area of the metal oxide increases.^30^ This indicates how the limitations and benefits of the composites are highly dependent on the constituents, their combination and the electrolyte which is beyond the scope of this study.

### 4.3 Rechargeable battery-type hybrid supercapacitors

The prospects of this category of supercapacitors lies in the struggle to rise through the midway diagonal of the Ragone plot which holds promising traits of higher specific capacitance, energy density and power density that still need to be excelled towards, compared to those of present supercapacitors. Some considered factors discussed here include surface modification, synthesis of a perfect nanocomposite material and microstructure optimization. As the creation of electroactive nanoparticles leads to faster reactions with the electrolyte, it should effectively lead to faster reactions, by undergoing redox with electroactive nanoparticles, as mentioned before in this study. However, a challenging matter arises because it is also possible that it will face spurious reactions with the electrolyte as well. In building nanocomposite materials with some metal oxides multiple challenges arise. One example of such a challenge lies with LiMnPO_4_, which has a higher potential than its Fe counterpart, then again are difficult to coat with a carbon layer like LiFePO_4_. But a remarkable strategic approach to overcome it was by creating a multi-layered structure of carbon layered over Fe over Mn, which did the trick. In fact, the study mentions that it performed better than expected at higher rates and without any direct contact of the oxidizing Mn metal oxide with the electrolyte itself.^31^ Electroactive species are prized for their valuable property in providing faster electrode reactions. One enhancing technique lies in developing granules in disproportioned fractions on the electrode surface. And so ‘fractal-granularity’, at the electrolyte–electrode interface, causes a higher surface area exposure leading to greater chances in raising the total energy that the capacitor provides. Another major improvement lies in applying the double layer concept to this type of supercapacitor. It creates a Helmholtz double layer where the charge is stored at the interface between the carbon electrode and the electrolyte. It occurs due to like charges being repelled from each other at the interface and attraction of counter ions, causing a physical charge storage mechanism to occur simply from the upsurge of polar ions. This had an effect of changing the energy density in this study by several orders of magnitude.

## 5. Applications of the ZnO semiconductor as an electrode material

Pseudocapacitors generally use reversible and fast reactions at the surface of electroactive (responsive to electrical stimuli) materials for charge storage. The large (typically from 300 up to 2000 F g^−1^) specific capacitance of faradaic electrodes exceeds that of carbonaceous materials using the electrical double layer charge storage mechanism, resulting in great attention towards these systems. Promising active pseudocapacitive materials include metal oxides such as NiO, RuO_2_, Fe_3_O_4_, MnO and ZnO. Yafei *et al.* prepared two distinct materials using a facile hydrothermal procedure: zinc–cobalt oxide and sulfide hybrid (ZCOSH) nanoclusters and compared them with zinc–cobalt binary oxide nanosheets (ZCO NSs). ZCOSH nanoclusters are designed by preparing a Ni foam, activated carbon electrodes and an NKK MPF30AC 100 membrane as a separator. The particle sizes of the samples are given roughly as 1 μm. The specific capacitance was recorded to be 2176.7 F g^−1^ for ZCOSH nanoclusters and 367.2 F g^−1^ for the ZCO NSs.^32^ Nanocrystalline (NC) hexagonal ZnO nanoflowers/reduced graphene oxide (ZnO-NFs/rGO) nanocomposite electrodes were prepared to test their electrochemical performance.^33^ The sample was prepared *via* direct chemical decomposition of zinc hexacyanoferrate (ZnHCF) over reduced graphene oxide (rGO) nanosheets. The electrode material consisted of ZnO-NFs/rGO nanocomposites, conductive carbon, a binder (PVDF) and methyl-2-pyrrolidone and the electrolyte was KOH solution. The particle size was approximately 1–2 μm and the specific capacitance was recorded to be 203 F g^−1^. Liu *et al.* studied the electrochemical performance of polyaniline/ZnO/Zeolitic Imidazolate Framework/graphene/polyester (PANI/ZnO/ZIF-8/G/P) as a fabric electrode material to build a solid state supercapacitor device. The electrode ZnO/ZIF-8/G/PC was prepared by chemical bath deposition and the electrolyte here is a gel material of PVA–H_2_SO_4_. The mean crystal size was not specified; however, their scanning electron microscopy (SEM) data revealed a 500 nm to 500 μm size range with a specific capacitance of 40 F g^−1^.^34^ Recently, scientists created ZnO nanoflake wrapped carbon nanofibers, ZnO/CNFs, using the electrospinning technique and hydrothermal process.^35^ It was used as an electrode material with an electrolyte of KOH solution. The specific capacitance was measured to be 260 F g^−1^. The composite nanowires had a diameter of 300 nm and lengths of several hundred micrometres giving a high aspect ratio. Yadav *et al.* investigated ZnO nanoparticles and their activated charcoal based nanocomposites as electrodes for electrochemical supercapacitors. They used the co-precipitation method to prepare the electrodes and used KOH and NaOH as the electrolyte. The sizes of the nanoparticles were calculated to be 30 nm and they have a specific capacitance of 341.6 F g^−1^.^36^ Also, the electrochemical deposition process has been used to build polypyrrole (Ppy)/graphene oxide (GO)/ZnO nanocomposites. The nanocomposites were deposited on nickel foam as electrodes which form a kind of sandwich with the polyvinyl alcohol hydrogel polymer as the electrolyte giving a specific capacitance of 123.8 F g^−1^. However, there was no mention of the sizes of the nanocomposites which can be understandable while it is a quite difficult task for such a design. Instead, 50 μm Ppy pore thickness was reported for this system.^37^ One of the effective methods to produce a ZnO/rGO nanocomposite as an electrode material is the combination of modified Hummers' method and ultrasonication. Using this smart technique 314 F g^−1^ specific capacitance and 5–10 nm particle sizes were recorded with the aid of KOH electrolyte.^38^ In another study, rGO/ZnO nanorods were prepared, sandwiched on both sides by polyethylene terephthalate (PET) substrate based thin film graphene rGO solution through chemical vapor deposition. The prepared nanorod samples of 50–100 nm diameter and 1 μm in length here revealed a value of 51.6 F g^−1^ specific capacitance with the help of KCl electrolyte.^39^ A particular nanocomposite consisting of ZnO particles decorated on carbon spheres was prepared to investigate its properties as an electrode, *via* a facile hydrothermal method and a low temperature water bath. It exhibited a specific capacitance of 630 F g^−1^ and a particle size of 106 nm, where KOH was used as an electrolyte.^40^ In another study, a graphene–zinc oxide (G–ZnO) nanocomposite was prepared with the aid of the solvothermal approach. With KOH operating as an electrolyte, the specific capacitance was recorded to be 122.4 F g^−1^ with nanocrystal sizes ranging between 30 and 70 nm.^41^ An electrode with a ZnO/MnO_2_ core–shell structure was prepared using Successive Ionic Layer Adsorption and Reaction (SILAR) deposition to build a planar supercapacitor. The diameter of the ZnO nanorods was found to be 200 nm. Na_2_SO_4_ was used as an electrolyte and the areal capacitance has been reported as 14 mF cm^−2^ (ref. 42) which is quite high. Additionally, while studying another sample, a ZnO activated carbon composite was synthesized *via* a precipitation reaction. The specific capacitance was found to be 155 F g^−1^. As the sizes of the particles were not of focus in this study, there was no mention of the sizes of the composite but only sample SEM images were provided. Na_2_SO_4_ was used as an electrolyte in this study.^43^ To build electrodes one step electrospinning and thermal treatment was used to make ZnO carbon nanofibers. The electrolyte used was KOH to measure their electrochemical properties, with a specific capacitance of 178.2 F g^−1^ and the diameter of Zn-ACNF (ZnO-containing porous activated carbon nanofibers) was recorded to be 200 nm.^44^ To test a sample made of carbon nanotube/graphite nanofiber/ZnO (CNT/GNF/ZnO), an electrode was synthesized using a hydrothermal process. The specific capacitance is measured to be 306 F g^−1^ with H_2_SO_4_ as an electrolyte. Although the exact size of the nanofibers is not explicitly mentioned, it shows in the study to be around 19.3 nm before ZnO deposition.^45^ Similarly, in another study, a hydrothermal process was also used to prepare a ZFO/rGO electrode and an electrolyte of a KOH solution. The particle sizes were measured to be around 15 nm and the specific capacitance was measured to be 352.9 F g^−1^.^46^Table 1 shows the details of the different electrodes succinctly and easily.

**Table 1:** Summarized traits of various ZnO nano-scale architectures used as electrodes for supercapacitors

Sample system	Specific capacity	Size	Capacitor type
Nanocluster	2176 F g^−1^	20 nm	Hybrid supercapacitor^32^
Nanoflower	203 F g^−1^	1–2 μm	EDLC and pseudocapacitor^33^
Fabric	40 F g^−1^	—	Hybrid supercapacitor^34^
Nanowire	260 F g^−1^	*d* = 300 *l* = 100 μm	Pseudocapacitor^35^
Nanoparticle	341.6 F g^−1^	32.30 nm	EDLC^36^
Nanocomposite	123.8 F g^−1^	—	EDLC^37^
Nanocomposite	314 F g^−1^	5–10 nm	Pseudocapacitor^38^
Nanorod	51.6 F g^−1^	*d* = 50–100 nm, *l* = 1 μm	EDLC and pseudocapacitor^39^
Nanosphere	630 F g^−1^	106 nm	EDLC^40^
Nanoparticle	160 F g^−1^	10–30 nm	Pseudocapacitor^47^
Nanocomposite	122.4 F g^−1^	30–70 nm	Pseudocapacitor^41^
Nanotube	347.3 F g^−1^	*d* = 40–60 nm *l* = 0.5–2 μm	EDLC and pseudocapacitor^48^
Nanorod	—	*d* = 200 nm	Pseudocapacitor^42^
Nanocomposite	155 F g^−1^	—	EDLC and pseudocapacitor^43^
Nanofiber	178.2 F g^−1^	*d* = 150 nm	EDLC and pseudocapacitor^44^
Nanotube	306 F g^−1^	*d* = 19.3 nm	EDLC and pseudocapacitor^45^
Nanoparticle	352.9 F g^−1^	15 nm	Pseudocapacitor^46^

## 6. Comparison of EDLCs and pseudo- and hybrid supercapacitors in terms of their specific capacitance

In a study to prepare EDLC electrodes, NiCo_2_O_4_ in MnO_2_ coresheet electrodes were designed and built by using a facile and stepwise hydrothermal approach. In testing this sample KOH electrolyte was used and the specific capacitance measured was 220 F g^−1^ and the thickness was 50 nm with dimensions of 2–3 μm for the nanosheets.^49^ Through the dissolution of zinc oxide nanoparticles, mesoporous activated carbon samples were prepared. They include three different ZnO weight samples, namely, Meso-AC10, Meso-AC20, and Meso-AC30 with specific capacitances of 152 F g^−1^, 164 F g^−1^ and 103 F g^−1^ respectively. KOF was used as an electrolyte and the nanopores have sizes that range between 2 and 50 nm.^50^ In another study, an electrode of zinc–cobalt layered double hydroxide was prepared through a hydrothermal approach. KOH was used as an electrolyte here and the calculated specific capacitance was 2142 F g^−1^. The nanowire structures had diameters of 50 nm and the lengths were mentioned to be uncertain.^51^ In a separate study, to prepare microporous activated carbon, thermal decomposition was used. Void spaces of 10–50 μm were mentioned; however, there was no mention of the sizes of the particles. The electrolyte used here was propylene carbonate solution. There was no mention of the specific capacitance of the activated carbon, and only its retention was measured by taking 0.1 A g^−1^ as a baseline.^52^ Another study used a tube furnace to prepare an electrode sample of polytetrafluoroethylene with carbon black. The electrolyte used was tetraethylammonium. The pore sizes ranged between 0.07 and 1.5 μm and the specific capacitance was measured to be 84.6 F g^−1^ for this nanocomposite.^53^ For building a carbon aerogel based supercapacitor, an electrode of Ni doped carbon aerogels was produced using catalytic reactions. The electrolyte was tested with acidic (H_2_SO_4_) and non-aqueous aprotic solutions (tetraethylammonium tetrafluorocarbonate) and the measured value of gravimetric capacitance was 182–219 F g^−1^ and 49–63 F g^−1^ respectively. The particle sizes are not mentioned; however the ion sizes were given as 0.70 nm, 0.46 nm, and 0.42 nm.^54^ Also, carbonization of natural alginic acid was used in creating a macro–meso–microporous prepared carbon aerogel (APCA). It was used as an electrode while KOH was used as an electrolyte. The specific capacitance was measured to be 188 F g^−1^ and the size of the nanopores was measured to be 200–300 nm in diameter.^51^ To prepare the desired supercapacitor of this category the drop-casting method was used to make a cyanographene nanoflake electrode with an aqueous solution of KOH as an electrolyte. It had lateral dimensions of about 0.5 μm and a specific capacitance of 191 F g^−1^.^55^ A special supercapacitor made of reduced graphene oxide (rGO)/amino functionalized CNT composites as electrodes was synthesized *via* the hydrothermal method. The electrolyte used was aqueous KOH. Its capacitance was measured to be 76.2 F g^−1^ and the diameters of CNT–NH_2_ were measured to be 3–2 nm (inner diameter) and 8–10 nm (outer diameter) using TEM.^56^ Moreover, using the hydrothermal process multiwall carbon nanotubes (MWNTs) decorated with zinc sulfide (ZnS) nanosheets were prepared. The specific capacitance was found to be 347.3 F g^−1^ and the carbon nanotubes were measured to have a diameter of 40–60 nm with a length of 0.5–2 μm. This also signifies a high aspect ratio. A polymer gel electrolyte was used in this all solid-state flexible supercapacitor.^48^ Electrodes made of TiCe CDC films were prepared using micro-fabrication processes including sputtering and photolithography. The thickness of the film was measured by TEM to be 1.6 μm and the areal capacitance was 1.4 mF cm^−2^. There was no mention of specific capacitance in F g^−1^. Tetraethylammonium tetrafluoroborate NEt_4_BF_4_, in propylene carbonate (PC), was used here as an electrolyte.^57^

Cationic poly(ionic liquid)s (PILs) are used as electrode–electrolyte materials to build pseudocapacitors. PIL1 copolymer used as an electrolyte made by free radical polymerization and PIL 2 as the electrode were prepared by quaternization and ion exchange with a metal salt. The specific capacitance was measured to be 150 F g^−1^ and the thickness of the electrode ranged between 0.8 and 24 μm.^58^ In another study, electrodes made of graphene beaded carbon nanofibers (G/CNFs) surface-coated with nanostructured conducting polymers were prepared by electrospinning and H_2_SO_4_ acted as the electrolyte. The electrode of G/CNF was measured to have 200–400 nm diameter possessing a specific capacitance of 637 F g^−1^.^59^ In a similar study to build pseudocapacitors, Li *et al.* prepared amine-enriched carbon electrodes prepared by the electrostatic fusion of amine-functionalized single-crystalline graphene quantum dots (GQDs) within TiO_2_ nanotubes. The specific capacitance was measured to be between 400 and 595 F g^−1^ when H_2_SO_4_ was used as an electrolyte and the nanotubes showed a length of 18 μm when carefully measured.^60^ To prepare metal oxide electrodes made of nanosized rambutan-like nickel oxide, a microwave-assisted method for fabrication was used with NaOH as an electrolyte. The specific capacitance of the electrodes was calculated to be 720 F g^−1^ and structures had a thickness less than 5 nm and a diameter of 250 nm.^61^ In another study, for creating metal oxide electrodes, Kang *et al.* prepared a nickel based metal organic framework (MOF), as an electrode *via* a hydrothermal reaction. The MOF is the positive electrode and activated carbon acts as a negative electrode in KOH electrolyte. It was measured to have a high specific capacitance of 726 F g^−1^. Emphasis was not was placed on the dimensions of the particle; therefore they were not included.^62^ Polymerization of aniline monomers was used to prepare the electrode and electrolyte polyaniline–rGO composite hydrogel. The supercapacitance was measured to be 712 F g^−1^ having nanoparticle sizes within 10–15 nm.^63^

In building hybrid supercapacitors, two distinct electrodes were prepared in one study: a microporous carbon as the negative electrode and a micro/mesoporous carbon as the positive electrode. The electrodes were designed using chemically activated carbon mixed with isopropanol, having no method specifically named for preparation. Electrolytes made of Li_2_SO_4_ with KI in aqueous solution were used while the pore sizes were measured to be 0.7–1.0 nm and the specific capacitance was 74 F g^−1^.^64^ In another study for preparing asymmetric hybrid capacitors, a precipitation synthesis technique was used to prepare carbon with Ni(OH)_2_ as a positive electrode and a Cu_2_O graphene composite as a negative electrode. KOH solution was used as an electrolyte. The specific capacity measured had increasing values ranging between 30 and 185 mA h g^−1^, though not mentioned in F g^−1^. The particle sizes were measured with TEM having a width of 100 nm and 1 μm length for the needle-like particles.^65^ In an effort to build another set of asymmetric electrodes a galvanostatic deposition method was used to prepare amorphous lead dioxide electrodes in this study. The electrolyte here was lead acetate and sodium acetate solution, and PbO_2_ was the positive electrode, whereas activated carbon was the negative electrode. The dimensions of PbO_2_ were around 1 μm and it provided a specific capacitance of 33 F g^−1^.^66^ To prepare composite hybrid supercapacitors Lee *et al.* prepared electrodes using a spray drying method where Li_4_Ti_5_O_12_ with activated carbon as an anode and activated carbon as a cathode with LiBF_4_ solution in ethylene carbonate and dimethyl carbonate as the electrolyte were used. The specific capacitance was measured to be 63 F g^−1^ with particle sizes ranging from 400 nm to 5 μm.^67^ Likewise, to prepare another set of composite hybrid electrodes, Sosreov *et al.* prepared electrodes made of nickel hydroxide and manganese hydroxide. They were tested in both pure KOH and LiOH–KOH as electrolytes. The small particle sizes ranged from 40 to 80 nm and the discharge capacitance is revealed to be 165 F g^−1^ (graph in 4c and 5c, reference).^68^ For this battery type supercapacitor, bilayered nickel hydroxide carbonate nanoplate-decorated nanoflowers were prepared using a facile homogeneous precipitation method. The areal capacitance was found to be 1445 mF cm^−2^ with no description of specific capacitance in F g^−1^. However the studies did show very small particle sizes of 2–4 μm when KOH was selected as the electrolyte.^69^ In another study, for also building this category of supercapacitors the researchers prepared Mo132–DTAB–EEG electrodes and a solution of aqueous H_2_SO_4_ electrolyte. The specific capacitance was measured to be 65 F g^−1^ and nanopore sizes were revealed to be within 4–5 nm. The samples were prepared using a binder, pressed on a Pt mesh and dried in a vacuum oven.^70^ While trying to build asymmetric pseudo-hybrid supercapacitors, Ni(OH)_2_ nanosheets were prepared using a centrifugation process to build the electrodes while using KOH as an electrolyte. The thickness was measured to be 40 nm and the combined system achieved a great capacitance of 934 F g^−1^.^71^ Meanwhile, to build electrodes for a similar type of capacitor another study used the one-pot hydrothermal process to prepare 15 nm thick nanosheets. They are made up the MoS_2_–Ni_3_S_2_ composite electrode with diluted PVA (polyvinyl alcohol) as an electrolyte. It remarkably displayed a high capacitance value of 1440.9 F g^−1^ (Table 2).^72^

**Table 2:** Properties of chosen supercapacitors from a recent research database

Sample system	Specific capacity	Size	Capacitor type
Nanosheet	220 F g^−1^	*t* = 50 nm, dimension = 2–3 μm	EDLC^49^
Nanoparticle	164 F g^−1^	2–50 nm	EDLC^50^
Nanowire	2142 F g^−1^	Diameter (*d*) = 50 nm	EDLC^51^
Nanoparticle	—	10–50 μm	Activated carbon EDLC^52^
Nanocomposite	84 F g^−1^	0.07–1.5 μm	Activated carbon EDLC^53^
Nanocomposite	219 F g^−1^ 63 F g^−1^	—	Carbon aerogel EDLC^54^
Nanoparticle	188 F g^−1^	200–300 nm	Carbon aerogel EDLC^73^
Nanoflake	191 F g^−1^	0.5 μm	Graphene pseudocapacitor^55^
Nanotube	76 F g^−1^	*d* = 8–10 nm	CNT EDLC^56^
Nanoparticle	—	1.6 μm	CDC EDLC^57^
Nanoparticle	74 F g^−1^	0.7–1 nm	Asymmetric hybrid supercapacitor^64^
Nanoparticle	—	1 μm	Asymmetric hybrid supercapacitor^65^
Nanoparticle	33 F g^−1^	1 μm	Asymmetric hybrid supercapacitor^66^
Nanocomposite	63 F g^−1^	400 nm to 5 μm	Composite hybrid supercapacitor^67^
Nanocomposite	165 F g^−1^	40–80 nm	Composite hybrid supercapacitor^68^
Nanoflower	—	2–4 μm	Battery-type hybrid supercapacitor^69^
Nanocomposite	65 F g^−1^	4–5 nm	Battery-type hybrid supercapacitor^70^
Nanosheet	934 F g^−1^	40 nm	Asymmetric pseudo/EDLC^71^
Nanosheet	1440 F g^−1^	15 nm	Asymmetric pseudo/EDLC^72^
Nanoparticle	720 F g^−1^	5 nm	Metal oxide pseudo-supercapacitor^61^
Nanocomposite	726 F g^−1^	—	Metal oxide pseudo-supercapacitor^62^
Nanocomposite polymer	712 F g^−1^	10–15 nm	Pseudocapacitor^63^
Copolymer	150 F g^−1^	—	Pseudocapacitor^58^
Nanostructured polymer	637 F g^−1^	200–400 nm	Pseudocapacitor^59^
Nanotube	595 F g^−1^	18 μm	Pseudocapacitor^60^

## 7. Conclusions

In this mini review, almost all types of supercapacitors were chosen to be analyzed in terms of their electrode materials, their designs, electrolyte and fabrication technique used along with their specific capacitance for a succinct depiction, conceptually reinforced with nice examples. This provides a connection to the specific capacitance values with the working principles, as exemplified in this article, for each type of supercapacitor along with nuances at the nanoscale that lead to their inherent structural and mechanical properties. To complete the portrayal in each category, favoured traits of high energy density, power density, high adsorption, and compressibility were balanced with imperfections or defects and challenges like poor porosity, low conductivity, imbalance at redox sites, degradation, rise in internal resistance, increase in equivalent series resistance *etc.* The mechanism of storage principles along with the categorization of supercapacitors has been summarized within this mini review work. The need for all kinds of supercapacitors can be justified due to the limitations of present energy harvesting and storage devices. It is obvious that each type of supercapacitor finds its applications as per their demand and their technological improvement. Especially, the applications of pseudo- and hybrid supercapacitors are growing particularly in the field of electric vehicles while most of the power consumption of the vehicles can be supplied by high-power supercapacitor devices. Additionally, a special section was devoted in this review to ZnO based supercapacitors due to their wide band gap, defective structure, and high electron mobility which is promising for high capacitive properties. As this is a mini review, the extent of our discussion about other character traits like the Ragone plot, charge–discharge rates and cyclability which are also critical in the design and choice of a capacitor has been curtailed. In a future study, we would like to delve into other key features including CV curves, TEM and SEM inspection, galvanostatic charge–discharge, and electrochemical impedance spectroscopy which are also invaluable for inspection of supercapacitors. The ultimate goal for supercapacitors or ultracapacitors both in research and industry is to obtain high power density and high energy density simultaneously. Such a difficult task can be accomplished not only by the smart design of capacitors but also by the development of electrodes, electrolytes and separator materials. Electrochemical performance test techniques also play a crucial role in investigating the device itself. Of course, this requires further fundamental understanding through both experimental and theoretical investigations. For the synthesis, design and optimization of new electrodes, smaller sizing of electrodes down to sub-nanometers, electrical and ionic conductivity, and mechanical and thermal stability, a better fundamental understanding by both theoretical and experimental work is highly required. It is crucial to fundamentally understand the working principles of electrode materials and the charge–discharge (intercalation–deintercalation) process in more realistic supercapacitor designs. This can be accomplished using both theoretical modelling at the electronic scale and experimental methods, in particular, involving advanced characterization techniques such as electron paramagnetic resonance spectroscopy and Raman spectroscopy, for instance, to detect the defect states or imperfections in electrodes. Therefore, technological developments in instrumentation together with materials science and engineering will enable scientists and engineers in the near future to produce next-generation robust high power capacitors which may be used as alternative energy storage and harvesting systems for electrical mobile systems such as electric vehicles, space solar panels and wind tribunes.

## Conflicts of interest

There are no conflicts to declare.

## Supplementary Material
